# Continuous Reassortment of Clade 2.3.4.4 H5N6 Highly Pathogenetic Avian Influenza Viruses Demonstrating High Risk to Public Health

**DOI:** 10.3390/pathogens9080670

**Published:** 2020-08-18

**Authors:** Huanan Li, Qian Li, Bo Li, Yang Guo, Jinchao Xing, Qiang Xu, Lele Liu, Jiahao Zhang, Wenbao Qi, Weixin Jia, Ming Liao

**Affiliations:** 1 National Avian Influenza Para-Reference Laboratory (Guangzhou), College of Veterinary Medicine, South China Agricultural University, Guangzhou 510642, China; huananli@stu.scau.edu.cn (H.L.); lzs_xmjxqsyz@yt.shandong.cn (Q.L.); boli1992@stu.scau.edu.cn (B.L.); guoxiaoxiaoyang@gmail.com (Y.G.); jinchaoxing100544@stu.scau.edu.cn (J.X.); xuqiang@stu.scau.edu.cn (Q.X.); leliu1994@stu.scau.edu.cn (L.L.); jiahaozhang@stu.scau.edu.cn (J.Z.); qiwenbao@scau.edu.cn (W.Q.); 2 Key Laboratory of Zoonosis, Ministry of Agriculture and Rural Affairs, Guangzhou 510642, China; 3 Guangdong Laboratory for Lingnan Modern Agriculture, Guangzhou 510642, China; 4 National and Regional Joint Engineering Laboratory for Medicament of Zoonosis Prevention and Control, Guangzhou 510642, China; 5 Key Laboratory of Zoonoses Prevention and Control of Guangdong Province, Guangzhou 510642, China; 6 Xiaqiu Animal Husbandry & Veterinary Station, Yantai 261400, China

**Keywords:** clade 2.3.4.4 H5N6, influenza virus, reassort, transmit, pathogenicity, pandemic

## Abstract

Since it firstly emerged in China in 2013, clade 2.3.4.4 H5N6 highly pathogenic avian influenza viruses (HPAIVs) has rapidly replaced predominant H5N1 to become the dominant H5 subtype in China, especially in ducks. Not only endemic in China, it also crossed the geographical barrier and emerged in South Korea, Japan, and Europe. Here, we analyzed the genetic properties of the clade 2.3.4.4 H5N6 HPAIVs with full genome sequences available online together with our own isolates. Phylogenetic analysis showed that clade 2.3.4.4 H5N6 HPAIVs continuously reassorted with local H5, H6, and H7N9/H9N2. Species analysis reveals that aquatic poultry and migratory birds became the dominant hosts of H5N6. Adaption to aquatic poultry might help clade 2.3.4.4 H5N6 better adapt to migratory birds, thus enabling it to become endemic in China. Besides, migratory birds might help clade 2.3.4.4 H5N6 transmit all over the world. Clade 2.3.4.4 H5N6 HPAIVs also showed a preference for α2,6-SA receptors when compared to other avian origin influenza viruses. Experiments in vitro and in vivo revealed that clade 2.3.4.4 H5N6 HPAIVs exhibited high replication efficiency in both avian and mammal cells, and it also showed high pathogenicity in both mice and chickens, demonstrating high risk to public health. Considering all the factors together, adaption to aquatic poultry and migratory birds helps clade 2.3.4.4 H5N6 overcome the geographical isolation, and it has potential to be the next influenza pandemic in the world, making it worthy of our attention.

## 1. Introduction

In 2013, clade 2.3.4.4 H5N6 HPAIVs emerged in China for the first time [1,2]. Since then, cases of clade 2.3.4.4 H5N6 have been identified in Laos, Vietnam, South Korea, and Japan [3,4,5,6], and it was also detected in Europe in 2017 [7]. Surveillance of Chinese live poultry markets even showed that H5N6 has become a dominant subtype in China, especially in aquatic poultry [8]. In China, H5N6 was found to not only transmit inter-species-wise between poultry and wild birds but also to infect mammals such as swine, felines, and even humans [9,10]. Clade 2.3.4.4 H5N6 not only has become endemic in China but also has the potential to be a world pandemic.

Apart from H5N6, HPAIVs of clades 2.3.2, 2.3.4, and 7.2 of H5 have cocirculated predominantly in domestic poultry, especially in aquatic poultry in China, since 2007 [11,12,13]. Low pathogenic avian influenza viruses (LPAIVs), such as H9N2 and H6Nx, have also circulated in the local poultry of China [14,15,16]. Even though LPAIVs showed low pathogenicity in avian species, H9N2 and H6N1 were found to infect humans and could cause mild clinical symptoms [17,18]. However, unlike the HPAIVs H5Nx, LPAIVs H9N2 and H6Nx have attracted less attention in public health studies. In 2013, novel reassortants H7N9 and H10N8 emerged in China and caused human infections and deaths [19,20]. Further studies revealed that all six internal genes of H7N9 and H10N8 originated from domestic, local H9N2 [21,22]. Genetic analysis also showed that poultry infected by H9N2 functioned as incubators for the emergence of many novel reassortant AIVs, which have the potential to cause human infections [23]. Studies on H6 in aquatic poultry reservoirs of South China revealed the evolutionary behavior of this influenza family in aquatic poultry, and H6 was introduced to other animal hosts by aquatic birds [16]. Since the first outbreak of H5N6 in 2013, it has become a dominant subtype in China, especially in domestic ducks [8]. As the genome of influenza virus is composed of segmented RNAs, reassortant influenza viruses would be generated during co-infection [24,25,26]. The dominant H5N6 in China exhibited a great gene diversity, and clade 2.3.4.4 H5N6 reassorted with H5N6, H6Nx and H7N9/H9N2 in China [8,27].

Since 2015, H5N6 has crossed geographical boundaries such as the Sea of Japan and emerged in Korea and Japan, causing a great economic loss to local poultry farming in these countries [5,6]. Not long after that, in 2017, H5N6 emerged in many European countries, such as the Netherlands, Switzerland, Greece, and the UK, and the H5N6 in Europe was the result of reassortment of local H5N8 [28]. The H5N6 isolates in these countries and regions exhibited a complex gene composition, and phylogenetic tree analysis revealed that these viruses were reassortant viruses. For the first time, in 2014, the HPAIV clade 2.3.4.4 H5N8, which first emerged in South Korea, crossed the geographical gap via migratory birds and spread rapidly and globally to Japan, China, Europe, and North America [29,30,31,32,33]. Furthermore, due to the continuous reassortment with local viruses, a novel H5N2 emerged on the circulation pathway of H5N8 and also spread in many regions, including Canada and America [33,34]. Now clade 2.3.4.4 H5N6 showed similar characteristics with the worldwide H5N2. 

While clade 2.3.4.4 H5N6 reassorted with other local influenza viruses, it might acquire gene segments or mutations more suitable for transmission. Some mutations in HA segment might change the binding ability between HA α-2,3 and α-2,6 glycans, which are common in avian species and mammalian species, respectively [35,36,37,38]. Besides, some mutations in internal genes might affect the virulence or transmission ability of influenza viruses, such as E627K, D701N, K526R, and A588V [39,40,41,42]. Taking all these findings into account, H5N6 also has the potential to transmit all over the world and may be the next world pandemic, which could be a new threat to world health.

In this study, we performed detailed genetic analysis of clade 2.3.4.4 H5N6, for the reassortments of H5N6 internal genes were unprecedentedly complex [43]. We also tested several biological characteristics of H5N6. The aim of our study is to better understand the transmission and pathogenicity of clade 2.3.4.4 H5N6. Our findings highlight clade 2.3.4.4 H5N6 as threat to public health and the next potential pandemic of the world.

## 2. Materials and Methods 

### 2.1. Ethical and Biological Safety Statement

All experiments related to HPAIVs were performed in an animal biosafety level 3 laboratory in compliance with approved protocols by the biosafety committee of South China Agriculture University.

All animals in the experiment were handled in accordance with the principles of the Basel Declaration and recommendations of the approved guidelines of the Experimental Animal Administration and Ethics Committee of South China Agriculture University (SCAUABSL2017-013; 13 June 2016). The protocol (SCAUABSL2017-013) was approved by the experimental Animal Administration and Ethics Committee of the South China Agricultural University.

### 2.2. Virus Isolation, RNA Extraction, RT-PCR, and DNA Sequencing

Swabs were collected from chickens, ducks, geese, swine, humans, and the environment. Virus isolation, RNA extraction, and sequencing were performed as previously described [22]. Briefly, all the specimens were propagated in 9–11-days-old specific pathogen-free, embryonated chicken eggs. RNA was extracted from HA-positive samples, and RT-PCR was conducted with universal primers reported by Hoffmann [44]. PCR products were purified and then sequenced. In conclusion, we totally isolated nine H5N6 viruses and sequenced the full genomes of them. Details about these viruses were in the supplementary materials (Table S1).

### 2.3. Phylogenetic Analyses

All the H5N6 of full genome sequences until 2019 were downloaded from GISAID (Global Initiative on Sharing All Influenza Data), GenBank (National Center for Biotechnology Information), and IRD (Influenza Research Database), and 974 strains were collected totally. Phylogenetic analysis work was performed on downloaded data, and full-genome sequences of isolated viruses were used. Multiple sequence alignments were performed using Muscle [45]. The maximum likelihood phylogenetic tree was constructed by IQ-TREE software (http://www.iqtree.org/) with the GTRGAMMA model [46]. Ultrafast bootstrap was also implemented, and 1000 replications were run [47].

### 2.4. Receptor Binding Specificity Assay

The receptor binding specificity was analyzed with a solid-phase binding assay. Briefly, plates were coated with serial dilutions of α2,3 glycans (Neu5Aca2-3Galb1-4GlcNAcb-PAA-biotin, 3’SLN, Glycotch) and α2,6 glycans (Neu5Aca2-6Galb1-4GlcNAcb1-PAA-Biotin, 6’SLN, Glycotch) overnight at 4 °C. Then, the glycan solution was removed, and the plates were blocked, washed, and incubated with a solution containing 26 hemagglutination units of influenza virus. The plates were incubated at 4 °C for 12 h. After being washed again, the plates were incubated with mouse monoclonal antibody against NP (Sino Biological). The plates were then washed again and incubated with horseradish peroxidase (HRP)-conjugated goat anti-mouse antibody. Finally, the plates were washed and incubated with 3,3’,5,5’-Tetramethylbenzidine (TMB) for 15 min at room temperature. The reaction was stopped with 0.1 M HCl, and the absorbance was read at 450 nM.

### 2.5. Growth Kinetics of Influenza in Different Cells

Chicken-origin fibroblast cells DF-1 with high prevalence of α2,3 glycans linked receptors [48], swine testicular-origin cells ST with mainly α2,3 glycans linked receptors [49], and human-origin alveolar basal epithelial cells A549 with both α2,3 glycans and α2,6 glycans linked receptors [50] were incubated at a multiplicity of infection (MOI) of 0.01 in 0.5 mL Dulbecco’s Modified Eagle Medium (DMEM) in 12-well plates. Following 1 hour of absorption, the medium was removed and washed with phosphate-buffered saline (PBS) three times. The well was then refilled with 1 mL DMEM containing 0.2% Bull Serum Albumin (BSA) and 0.2 µg/mL tosyl-phenylalanine chloromethyl-ketone (TPCK) treated trypsin for DF-1 and ST and 0.2% BSA and 0.5 µg/mL TPCK treated trypsin for A549. Supernatants were collected at 12, 24, 36, 48, and 60 hours post-infection (hpi) and stored at −80 °C. The 50% tissue culture infectious dose (TCID_50_) of different samples were determined in MDCK cells.

### 2.6. Mice and Chickens Challenge Studies

Four-week-old female specific pathogen-free (SPF) BALB/c mice were divided into four groups randomly, 12 mice per group. Mice were inoculated intranasally with 10^6^ 50% egg infectious doses (EID_50_) of test viruses or sterile DMEM in a volume of 50 µL. Mice were monitored daily for weight loss over a period of 14 days. Three mice from each group were euthanized at 3 days post-infection (dpi), and lung and brain samples were collected for virus titration. The mice whose weight loss was more than 25% were also humanely euthanized. The mice were euthanized by CO_2_.

A total of 27 six-week-old SPF chickens were divided into three groups, nine chickens per group, and then were inoculated intranasally with 10^6^ EID_50_ of test viruses in a volume of 200 µL. Non-inoculated hatch-mates were added to each group at 1 day post-infection (dpi, contacts). Six contact chickens for 673 and 674 group and nine contact chickens for 39715 group. Clinical signs were monitored daily until 14 dpi. At 2, 3, and 5 dpi, both cloacal and throat swabs were collected. Chickens were humanely euthanized by cervical dislocation at 2, 3 and 5 dpi. The organs including heart, liver, spleen, kidney, brain, and lung of the chickens succumbing to viral infection were also collected. All the swabs and organs were titrated by embryo eggs. The same actions were also performed on naive contact groups.

The virus titer of mice and chickens were tittered in embryonated eggs. The hemagglutination activity assay was performed by 1% chicken red blood cells.

## 3. Results

### 3.1. Phylogenetic Analysis of Surface Genes of H5N6 Viruses

**Figure 1:**
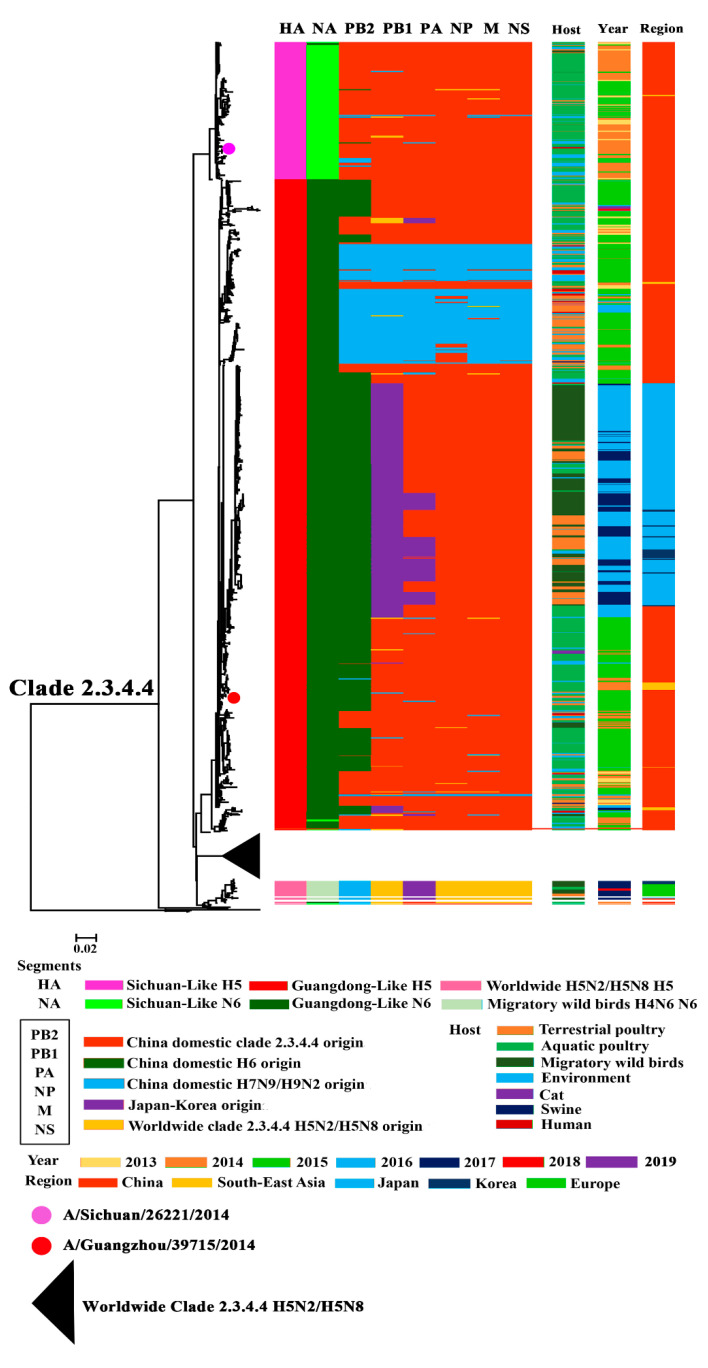
Phylogenetic analysis of H5N6 influenza viruses.

To make a better understand of the establishment and evolution of H5N6, we performed a phylogenetic analysis of all the H5N6 genomes available from GISAID, GenBank, and IRD, together with H5N6 viruses isolated by our lab. The phylogeny analysis result of HA showed that nearly all the H5N6 belonged to clade 2.3.4.4, with only a few exceptions (Figure 1, Figure S1). The HA of clade 2.3.4.4 H5N6 could be divided into three groups: Sichuan-Like (SC-Like), Guangdong-Like (GD-Like), and Worldwide clade 2.3.4.4 H5N2/H5N8-Like (World-H5-Like) (Figure 1). The SC-Like and GD-Like showed nucleotide similarity to human-origin isolates A/Sichuan/26221/2014(H5N6) and A/Guangzhou/39715/2014(H5N6), respectively. The World-H5-Like showed higher identity with the H5N2 and H5N8 HPAIVs, which spread globally. GD-Like viruses took a major part of all the clade 2.3.4.4 H5N6 HPAIVs.

The phylogeny of NA revealed that the NA of clade 2.3.4.4 H5N6 HPAIVs could be divided into three lineages: Sichuan-Like (SC-Like), Guangdong-Like (GD-Like), and H4N6-Like (Figure 1, Figure S2). The NA segment of both SC-Like and GD-like viruses exhibited similarity to H6N6 circulating in domestic poultry of China; however, the NA of H4N6-Like viruses is similar to the H4N6 of migratory birds. All the NA genes of SC-Like and GD-Like viruses, according to nucleotide similarity to human-origin isolates A/Sichuan/26221/2014(H5N6) and A/Guangzhou/39715/2014(H5N6), respectively, belonged to ST-192-Like, which was the major lineage of H6N6 circulating among aquatic poultry in China [16]. Furthermore, nearly all the GD-Like NAs had a stalk deletion in the stalk region, with only few exceptions, while the SC-Like and H4N6-Like viruses all harbored a full-length NA without a stalk deletion.

### 3.2. Phylogenetic Analysis of Internal Genes of Clade 2.3.4.4 H5N6 HPAIVs

When constructing the phylogenetic trees of internal genes, we considered that bootstrap values of > 95% were reliable. The internal genes of clade 2.3.4.4 H5N6 exhibited great diversity. We found that PB2 could be mainly classified into three lineages, which originated from China domestic clade 2.3.4 H5, China domestic H6, and China domestic H7N9/H9N2. PB1 could be divided into four lineages, including China domestic clade 2.3.4 H5 origin lineage, China domestic H7N9/H9N2 lineage, Japan–Korea lineage, and Worldwide clade 2.3.4.4 H5N2/H5N8 lineage. The PA gene of clade 2.3.4.4 H5N6 originated from China domestic clade 2.3.4.4 H5, H7N9/H9N2 internal gene and the Japan–Korea lineage. Segments NP, M, and NS had the same source and were provided by China domestic clade 2.3.4 H5, China domestic H7N9/H9N2, and Worldwide H5N2/H5N8.

Further analysis of internal genes revealed that the H7N9/H9N2 origin genes showed a district characteristic: gene segments from the same province shared a higher nucleotide identity (Supplementary Figures S3–S8) [8]. This phenomenon means that H5N6 might keep reassorting with local chicken H7N9/H9N2. According to Huang’s work on H6 in China, the H6 lineage PB2 belonged to Group I/II of China domestic H6 PB2 [16]. The Japan–Korea lineage PB1 and PA fell into the gene pool of China domestic H6. Furthermore, both Group I/II and gene pool H6 provided genes for H6 circulating in aquatic poultry [16]. These segments might make H5N6 more adapted to aquatic poultry and migratory birds. Additionally, while circulating all over the world, H5N6 also reassorted with the worldwide H5N2/H5N8 carried by migratory birds and local poultry. This was why H5N6 also harbored segments originating from H5N2/H5N8. We also found that the internal genes of H5N6 exhibited a great diversity, and this might help H5N6 cross the inter-species barrier, suggesting its potential threat to world health.

### 3.3. Continuously Reassortment of Clade 2.3.4.4 H5N6 HPAIVs

**Figure 2:**
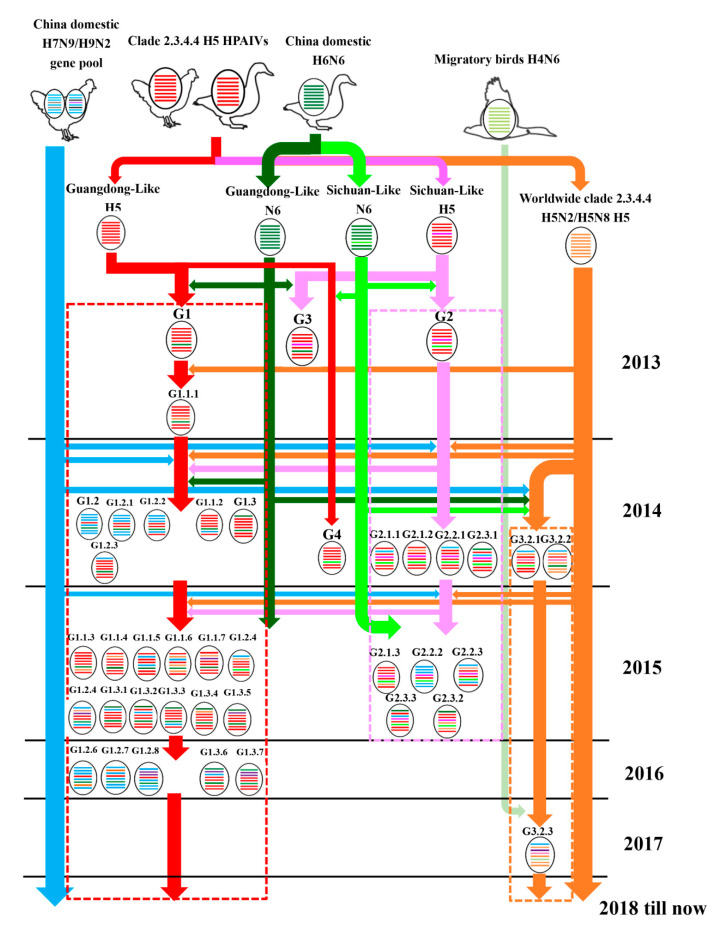
Evolutionary pathway of clade 2.3.4.4 H5N6 HPAIVs. The eight gene segments are PB2, PB1, PA, HA, NP, NA, M, and NS (from top to bottom of the virion). Different colors represent different virus lineages.

We found an interesting phenomenon that there were particular HA:NA combinations of clade 2.3.4.4 H5N6. Most of clade 2.3.4.4 H5N6 belonged to a GD-Like:GD-Like combination, an SC-Like:SC-Like combination, or a World-H5-Like:H4N6-Like combination. Only a few viruses were excepted (Figure 1 and Figure 2).

A previous study by our group showed that the GD-Like:GD-Like H5N6 exhibited enhanced pathogenicity and transmissibility in chickens as compared to other H5Nx subtypes and H5N6 of other HA-NA combinations [51,52]. It was revealed that the appropriate match of HA and NA promoted the GD-Like:GD-Like combination and became the dominant H5N6 genotype in China. Over time, only the GD-Like:GD-Like combination and World-H5-Like:H4N6-Like combination were left. These combinations might enhance H5N6 replication and transmission.

### 3.4. Host, Time, and Region Distribution of Clade 2.3.4.4 H5N6 HPAIVs

**Figure 3:**
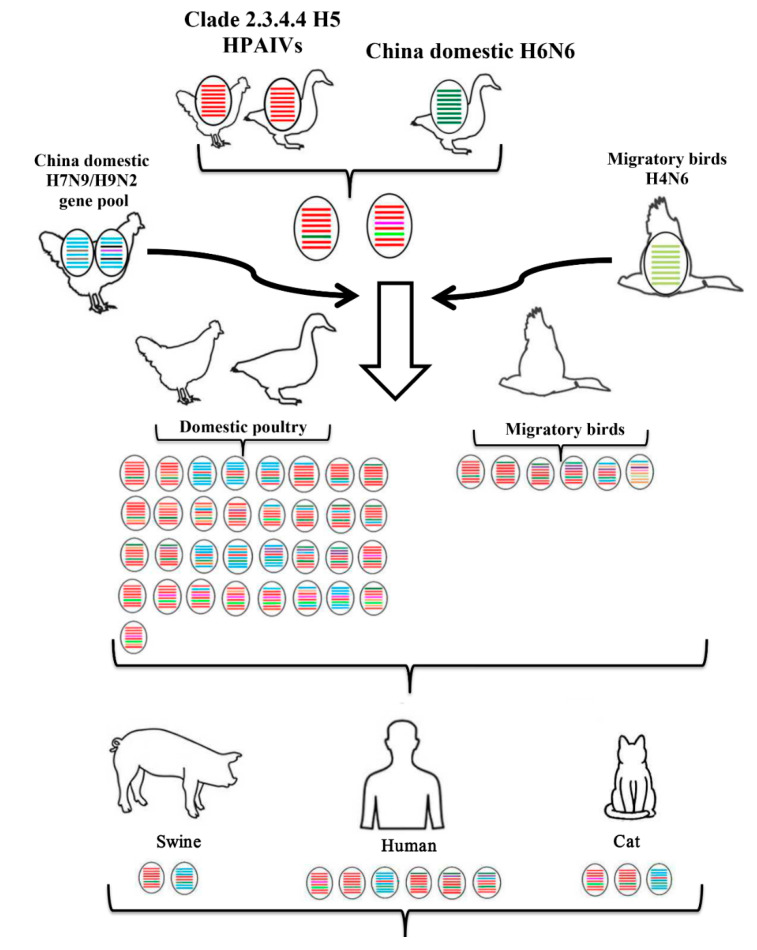
Host of clade 2.3.4.4 H5N6 HPAIVs. The eight gene segments are PB2, PB1, PA, HA, NP, NA, M, and NS (from top to bottom of the virion). Different colors represent different virus lineages.

The conclusion of our summary work shows that the primary hosts of H5N6 were aquatic poultry (ducks and geese) and migratory birds, even though it has a wide host range (Figure 1 and Figure 2). It is possible that the adaption to aquatic poultry and migratory birds of H5N6 was the principal reason why the virus could move over long distances and spread globally. The gene composition of H5N6, especially the internal genes, became more and more complex as time went on (Figure 2). This revealed the continuous reassortment between H5N6 and other subtype viruses and reassortment between different H5N6 viruses. Additionally, there was also a clear spatio-temporal correlation. First, H5N6 emerged in China and South Asia in 2013. Then it transmitted to South Korea and Japan in 2016. In 2017, H5N6 isolates were reported in Europe. The time clue of evolution also showed that clade 2.3.4.4 H5N6 spread continuously and unrestrictedly (Figure 2). The hosts of H5N6 also exhibited a diversity. Except domestic poultry and migratory birds, swine, cats, and humans were also found to be infected with H5N6 (Figure 3). The gene composition of viruses isolated from swine, cats, and humans also showed a multiplicity of sources (Figure 3). It reminds us that clade 2.3.4.4 H5N6 HPAIVs has a great potential to infect humans.

### 3.5. Receptor Binding Ability of Clade 2.3.4.4 H5N6 HPAIVs

HA of influenza virus was considered to be related with the host range, pathogenicity, and transmissibility in avian and mammalian species.

**Figure 4:**
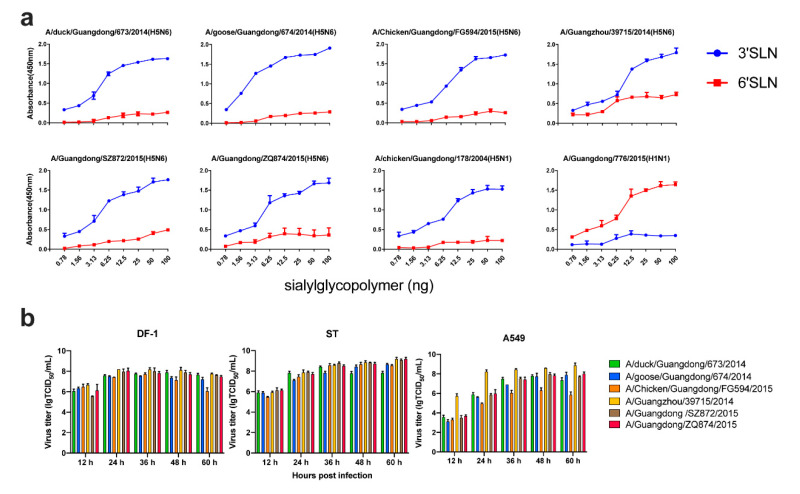
Receptor binding ability and growth curve of clade 2.3.4.4 H5N6 HPAIVs. (**a**) Receptor reference of clade 2.3.4.4 H5N6 HPAIVs. A 2009 pandemic H1N1 virus (A/Guangdong/776/2015) and a clade 2.3.2 H5N1 virus (A/Guangdong/178/2004) were used as positives controls for SA2,6Gal receptors and SA2,3Gal receptors, respectively. (**b**) Virus titer of H5N6 AIVs on different cell lines. DF-1, ST, and A549 were inoculated at a MOI of 0.01; supernatants were collected at 12, 24, 36, 48, and 60 hours post-infection (hpi) and stored at -80 °C. The 50% tissue culture infectious dose (TCID_50_) of different samples were determined in MDCK cells.

After analyzing all the HA sequences of clade 2.3.4.4 H5N6 available, we found that they all contained a Q226/G228 (H3 numbering). This means that clade 2.3.4.4 H5N6 might show a preference to α2,3-SA. To better understand the receptor binding preference of clade 2.3.4.4 H5N6, we ran a solid-phase binding assay with plates coated with SA2,3Gal and SA2,6Gal sialylglycopolymers. The 2009 pandemic H1N1 virus (776) and a clade 2.3.2 H5N1 virus (178) were used as controls. All the H5N6 viruses exhibited a preferential binding to SA2,3Gal receptors (Figure 4a). However, the human isolate 39715 exhibited a higher binding ability to SA2,6Gal receptors compared to clade 2.3.2 H5N1 virus.

### 3.6. Replication of Clade 2.3.4.4 H5N6 in Different Cells

To assess the replication ability of clade 2.3.4.4 H5N6 in different cells, we performed a multistep growth curve of several viruses isolated from different hosts in DF-1, ST, and A549 cells. All the viruses replicated efficiently in these three kinds of cells, which could reach a high titer of >7 lg TCID50/mL (Figure 4b). Even though different genotypes of clade 2.3.4.4 H5N6 had different origin gene segments, they all replicated very well in avian cells, swine cells, and human cells. This finding indicates that clade 2.3.4.4 H5N6 should be considered as a risk for public health.

### 3.7. Pathogenicity of Clade 2.3.4.4 H5N6 in Mice

To assess the pathogenicity of Clade 2.3.4.4 H5N6 in mice, mice were inoculated intranasally with 10^6^ EID_50_ of A/duck/Guangdong/673/2014 (SC-Like HA:SC-Like NA; Clade 2.3.4 origin internal genes), A/goose/Guangdong/674/2014 (GD-Like HA; GD-Like NA; Clade 2.3.4 origin internal genes), and A/Guangzhou/39715/2014 (GD-Like HA; GD-Like NA; Clade 2.3.4 origin internal genes).

**Figure 5:**
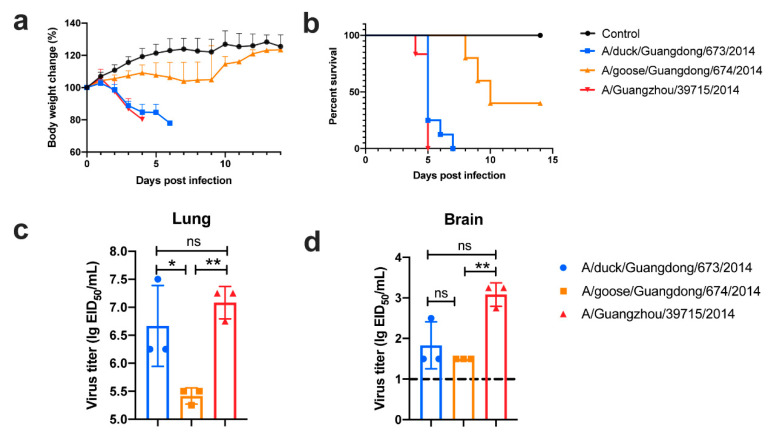
Four-week-old female specific pathogen-free (SPF) BALB/c mice were inoculated intranasally with 10^6^ egg infectious doses (EID_50_) of test viruses or sterile Dulbecco’s Modified Eagle Medium (DMEM) in a volume of 50 µL. Body weight change (**a**) and survival rate (**b**) of mice were exhibited. The virus load of lung (**c**) and brain (**d**) of infected mice were determined on 3 dpi. The dash line represented the detection limit of this assay. For statistical analysis, a value of 1.5 was assigned if the virus was not detected from the undiluted sample in three embryonated eggs. The data was analyzed in one-way ANOVA test by GraphPad. * meant *p* < 0.05, ** meant *p* < 0.01, and ns meant no significant difference, respectively.

All the selected viruses were lethal to mice, and 673 and 39715 could cause a lethality of 100% (Figure 5a,b). All three viruses were capable of replication in the lungs of inoculated mice and could reach as high as 7.25 lg EID_50_/mL (Figure 5c). Additionally, 673 and 39715 could replicate in the brains of inoculated mice; however, 674 was replication-deficient in this organ (Figure 5d).

This revealed that clade 2.3.4.4 H5N6 can replicate in mammals and exhibits a lethal risk. In fact, 673 could reach a lethality of 100% in mice, while 674 showed a lethality of less than 60%. H5N6 viruses like 673 (SC-HA:SC-NA) cause sudden death to mammalian hosts, diminishing the transmission ability to some extent. However, viruses similar to 674 (GD-HA:GD-NA) are not 100% lethal to mammalian hosts and can also replicate in the lungs of infected animals. Viruses similar to 674 could replicate in the respiratory organs of infected mice but show mild pathogenicity to mice. Additionally, these could achieve some mammalian adaption mutation in the internal genes. The gene composition of 39715 is similar to 674, but 39715 exhibited a high pathogenicity to mice. Mammalian adaption mutations such as E627K or D701N are also found in human isolates. One of reasons why 39715 exhibited a high pathogenicity to mice is that 39715 has the E627K mutation in PB2 (Table S2). This might be one of the reasons why nearly all the human isolates of H5N6 harbor a GD-Like HA and GD-Like NA like 674.

### 3.8. Pathogenicity of Clade 2.3.4.4 H5N6 in Chickens

To investigate the virulence and transmission ability of these viruses in chickens, SPF chickens were inoculated intranasally with 10^6^ EID_50_ of selected viruses in a volume of 200 µL.

All the selected viruses caused a lethality of 100% to inoculated chickens in three days and were capable of replication in all the organs, including lung, heart, liver, spleen, kidney, and brain (Table 1). The virus titers in hearts, livers, spleens, kidneys, brains, and lungs were quite high; for example the virus titer in lung could reach > 6 lg EID_50_/mL, and even the virus titer in brain could reach > 5 lg EID_50_/mL. Even though clade 2.3.4.4 H5N6 exhibited a high virulence to chickens, the transmission ability was quite different (Table 2). Contact groups 674 and 39715 shed viruses through throat and cloaca, but 673 contact group only shed viruses at 2 dpi through cloaca. This phenomenon means the transmission ability of 674 and 39715 is stronger than that of 673 in chickens. This should explain why GD-Like HA and GD-Like NA matches became the dominant matches of clade 2.3.4.4 H5N6.

## 4. Discussion

In recent years, HPAIVs H5Nx such as H5N2, H5N8, and H5N6 have emerged and replaced predominant H5N1 and became the dominant subtypes. The newly emerged HPAIVs clade 2.3.4.4 H5N6 spread rapidly in South and Central China, replacing H5N1 to become the dominant H5 subtype in China. It is notable that South China and Central China shared the highest aquatic poultry feeding density in China. The human infection cases also took place in these areas. No isolates were reported in other inland provinces with limited waterflow production. Therefore, we hypothesize that aquatic poultry helped H5N6 spread and became endemic in China.

Compared to predominant H5N1 and the worldwide H5N2/H5N8, the newly emerged H5N6 has a more complex and more diverse gene segment composition. Clade 2.3.4.4 HA could be divided into three groups. The H6N6 origin N6 also fell into two groups. During the study of surface genes of H5N6, we found that the match of GD-Like HA with GD-Like NA was significant in China and became the dominant surface gene composition over time. The internal genes of H5N6 were more complex when compared to the surface genes. Further phylogenetic analysis of the internal gene of clade 2.3.4.4 H5N6 viruses in China revealed that the internal genes were similar to H5, H6, and H7N9/H9N2 viruses (Figures S2–S8), which were the three most common AIVs circulating in the poultry of China. The H5 origin genes all fell into clade 2.3.4, which was a dominant clade in China [53]. Previous research also revealed that clade 2.3.4 AIV could induce severe inflammatory responses in human immune cells compared to clade 2.3.2 and clade 7.2, two other dominant clades in China [54]. Therefore, H5N6 might present a higher risk for public health. H7N9/H9N2 origin internal genes may stem from local poultry because we also discovered an obvious regional signature of these genes. For example, isolates from the same province showed a higher identity (Figures S3–S8); however, H5 origin and H6 origin genes did not have this phenomenon. The H6 origin internal genes were found to be provided by the aquatic poultry of South China. The H5N6 spread to South Korea and Japan all had H6 origin segments. We do believe that H6 origin segments helped H5N6 adapt better to aquatic poultry and migrant birds, allowing it to cross the ocean barrier and spread to South Korea and Japan. As previous findings revealed, HPAIVs H5N2 and H5N8 were spread all over North America, and HPAIV H5N8 circulated in Europe. H5N6 also has the potential to spread globally. Even though the European H5N6 isolates harbored surface genes different from the predominant H5N6 of China, the internal genes shared some identical segments with the H5N6 of China. Compared to the local H5N2/H5N8, the European isolates harbored H7N9/H9N2 lineage origin PB2 and Japan–Korea lineage origin PA. The internal genes of H9N2 were considered as a key factor for the circulation of H7N9 in China [21], and they also played a role in the recent emergence of human H10N8 infections [22]. Up to now, there are no reports about worldwide human infection cases of H5N2/H5N8. However, the European H5N6 harboring H7N9/H9N2 origin segments exhibits a potential threat to human health. The Japan–Korea origin segment might enhance the transmission ability of viruses in migratory birds and aquatic poultry. This highlights the potential of H5N6 to be spread globally and even be a world pandemic. Clade 2.3.4.4 H5N6 HPAIV is capable of replication in avian, swine, and human cells, and it is lethal to both mice and chickens [53,54]. It therefore exhibits a high risk to public health.

Furthermore, aquatic poultry was thought to be a natural reservoir of AIVs. However, not enough attention has been paid to it. Our team insists that the precautions taken regarding AIVs should be moved forward to the surveillance of aquatic poultry. We have invented the H5 vaccines particular for aquatic poultry, D7 and rD8, which have been approved by the Ministry of Agriculture and Rural Affairs of China. More attention should be paid to the AIVs circulating in aquatic poultry.

All the findings combined with our results reveal that clade 2.3.4.4 H5N6 HPAIV shows a preference for migratory birds and aquatic poultry and exhibits great genetic diversity. With the adaption to migratory birds and aquatic poultry, H5N6 has been transported over long distances. During the long-distance transmission of H5N6, it also kept continuously reassorting with local AIVs. Taking all the findings into consideration, H5N6 shows the potential for global spread and poses a great threat to the poultry industry and human health.

## Figures and Tables

**Table 1:** Virus titer of different organs (lgEID_50_/mL) ^a^.

Virus		Heart	Liver	Spleen	Kidney	Brain	Lung	Time of Death
673	inoculated	3/3(6.70 ± 0.30)	3/3(6.25 ± 0.25)	3/3(6.20 ± 0.10)	3/3(6.70 ± 0.02)	3/3(5.60 ± 0.02)	3/3(7.00 ± 0.40)	2d ^b^
	contact	0/2(0)	0/2(0)	0/2(0)	0/2(0)	0/2(0)	0/2(0)	4d ^c^
674	inoculated	3/3(6.70 ± 0.30)	3/3(6.00 ± 0.20)	3/3(6.50 ± 0.06)	3/3(6.40 ± 0.08)	3/3(5.30 ± 0.50)	3/3(6.70 ± 0.30)	2d ^b^
	contact	2/2(6.50, 7.25)	2/2(6.50, 6.25)	2/2(6.25, 7.25)	2/2(6.25, 7.25)	2/2(5.75, 7.25)	2/2(6.50, 8.50)	4d ^b^
39,715	inoculated	2/3(6.75, 7.50)	2/3(5.50, 6.25)	2/3(4.75, 6.50)	2/3(6.25, 7.50)	2/3(6.50, 7.25)	3/3(7.25 ± 1.19)	1/2d, 2/3d ^b^
	contact	0/3(0)	0/3(0)	0/3(0)	0/3(0)	0/3(0)	2/3(1.50, 3.75)	4d ^b^

^a^ Virus titers in hearts, livers, spleens, kidneys, brains, and lungs of the first three dead chickens or of the naive contact chickens on 4 dpi. ^b^ The chickens were succumbing to viral infection. ^c^ The chickens were euthanized.

**Table 2:** Virus titer and isolations (lgEID_50_/mL) ^a.^

Virus		Throat Swab ^b^			Cloacal Swab ^b^			Death Amount ^c^						
		2d	3d	5d	2d	3d	5d	2d	3d	4d	5d	7d	8d	10d
673	inoculated	3/3 (2.60 ± 0.3)	---d	---d	1/3 (1.75)	---d	---d	8/9	1/9	---e	---e	---e	---e	---e
	contact	0/6 (0)	0/6 (0)	0/4 (0)	2/6 (1.75, 2.50)	0/6 (0)	0/4 (0)	0/6	0/6	2/6	1/6	0/6	0/6	0/6
674	inoculated	1/1 (4.5)	---d	---d	1/1 (3.25)	---d	---d	9/9	---e	---e	---e	---e	---e	---e
	contact	0/6 (0)	2/6 (2.25, 3.75)	---d	0/6 (0)	1/6 (2.25)	---d	0/6	0/6	3/6	3/6	---e	---e	---e
39,715	inoculated	2/8 (1.75, 2.75)	4/8 (3.2 ± 0.09)	0/2 (0)	2/8 (1.50, 3.50)	4/8 (2.4 ± 0.8)	0/2 (0)	1/9	3/9	1/9	2/9	1/9	0/9	1/9
	contact	2/7 (1.50, 3.50)	3/7 (1.50 ± 0.06)	2/4 (1.25, 3.50)	0/7 (0)	1/7 (1.25)	3/4 (2.80 ± 1.3)	0/9	0/9	3/9	1/9	1/9	1/9	1/9

^a^ Six-week-old SPF chicken were inoculated intranasally with 10^6^ EID50 of test viruses in a volume of 200 µL. Cloacal and throat swabs were collected on 2, 3, and 5 dpi. from infected and naive chickens. ^b^ x/y meant positive/living total. ^c^ x/y meant death amount/total. ^d^ The chickens that died out at the sampling time point. ^e^ The chickens have died out.

## Supplementary Materials

The following are available online at https://www.mdpi.com/2076-0817/9/8/670/s1, Figure S1: Phylogenetic tree of HA, Figure S2: Phylogenetic tree of NA, Figure S3: Phylogenetic tree of PB2, Figure S4: Phylogenetic tree of PB1, Figure S5: Phylogenetic tree of PA, Figure S6: Phylogenetic tree of NP, Figure S7: Phylogenetic tree of M, Figure S8: Phylogenetic tree of NS, Table S1: Basic information of viruses used in this study, Table S2: Key molecular markers of viruses used in this study.
