# Supplementary material for: Continuous Reassortment of Clade 2.3.4.4 H5N6 Highly Pathogenetic Avian Influenza Viruses Demonstrating High Risk to Public Health
Source: Pathogens. 2020 Aug 18;9(8):670. doi: 10.3390/pathogens9080670 (PMC7460007; doi:10.3390/pathogens9080670)
Supplement: Supplementary file 1 [file pathogens-09-00670-s001.zip › Supplementary materials/Supplementary Files/supplementary materials Table S1-Table S2.docx]

**Table S1.** Basic information of viruses used in this study

|  | **Virus** | **Accession**  **No.** | **Host** | **Gene composition** | | | | | | | |
| --- | --- | --- | --- | --- | --- | --- | --- | --- | --- | --- | --- |
|  |  |  |  | **HA** | **NA** | **PB2** | **PB1** | **PA** | **NP** | **M** | **NS** |
| 1 | A/duck/Guangdong/673/2014 | EPI_ISL_259923 | Duck |  |  |  |  |  |  |  |  |
| 2 | A/duck/Guangdong/674/2014 | EPI_ISL_259924 | Duck |  |  |  |  |  |  |  |  |
| 3 | A/Guangzhou/39715/2014 | EPI_ISL_175335 | Human (Survived) |  |  |  |  |  |  |  |  |
| 4 | A/Guangdong/SZ872/2015 | EPI_ISL_206568 | Human (Dead) |  |  |  |  |  |  |  |  |
| 5 | A/Guangdong/ZQ874/2015 | EPI_ISL_206569 | Human (Survived) |  |  |  |  |  |  |  |  |
| 6 | A/swine/Guangdong/C135/2015 | No upload | Swine |  |  |  |  |  |  |  |  |
| 7 | A/swine/Guangdong/C136/2015 | No upload | Swine |  |  |  |  |  |  |  |  |
| 8 | A/duck/Guangdong/Xinyi/2018 | No upload | Duck |  |  |  |  |  |  |  |  |
| 9 | A/quail/Guangdong/HYF/2018 | No upload | Quail |  |  |  |  |  |  |  |  |


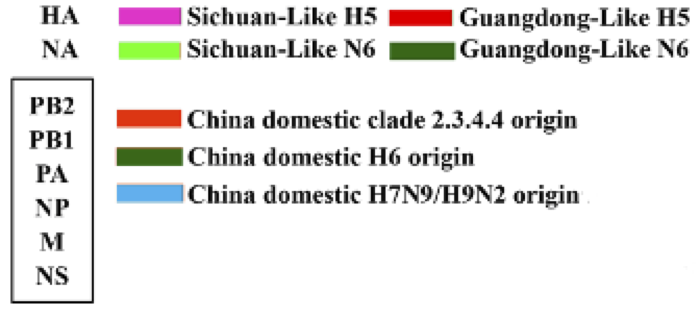


**Table S2.** Key molecular markers of viruses used in this study

| Virus | HA | | | | | | |  | NA | | | |  | PB2 | | |  | M2 |  | NS1 | |
| --- | --- | --- | --- | --- | --- | --- | --- | --- | --- | --- | --- | --- | --- | --- | --- | --- | --- | --- | --- | --- | --- |
|  | Cleavage site | RBS | | | | | |  | Deletion | 222 | 274 | 292 |  | 588 | 627 | 701 |  | 31 |  | Deletion | 92 |
|  |  | 158-160 | 187 | 193 | 196 | 133-137 | 225-228 |  | 59-69 |  |  |  |  |  |  |  |  |  |  | 80-84 |  |
| Clade  2.3.4  H5N1 | RERRRKR↓G | NNT | D | K | Q | SGVSS | GQSG |  |  |  |  |  |  |  |  |  |  |  |  |  |  |
| 673 | REKRRKR↓G | NDA | N | N | N | LGVSA | GQRG |  | No | I | H | R |  | T | E | D |  | S |  | Yes | E |
| 674 | RERRRKR↓G | NDA | N | N | N | LGVSA | GQRG |  | Yes | I | H | R |  | T | E | D |  | S |  | Yes | E |
| 39715 | RERRRKR↓G | NDA | N | N | N | LGVSA | GQRG |  | Yes | I | H | R |  | A | K | D |  | S |  | Yes | E |
